# A Pan-Cancer Analysis of IRAK1 Expression and Their Association With Immunotherapy Response

**DOI:** 10.3389/fmolb.2022.904959

**Published:** 2022-05-20

**Authors:** Mengmeng Liu, Yi Que, Ye Hong, Lian Zhang, Xing Zhang, Yizhuo Zhang

**Affiliations:** ^1^ Sun Yat-sen University Cancer Center, State Key Laboratory of Oncology in South China, Collaborative Innovation Center for Cancer Medicine, Guangzhou, China; ^2^ Department of Medical Melanoma and Sarcoma, Sun Yat-sen University Cancer Center, Guangzhou, China; ^3^ Department of Pediatric Oncology, Sun Yat-sen University Cancer Center, Guangzhou, China

**Keywords:** IRAK1, pan-cancer analysis, PD-L1, prognosis, immunotherapy

## Abstract

IRAK1 is an active kinase which plays a critical role in IL-1/TLR signaling pathway involved in inflammation and innate immune response. Recently, increasing evidence supports a potential role of IRAK1 in cancer progression. However, no immunological pan-cancer analysis of IRAK1 is available. We aimed to explore the prognostic value and the immunological functions of IRAK1. A series of datasets including The Cancer Genome Atlas, GEPIA2, cBioPortal, HPA, TIMER2.0 were performed to explore the oncogenic and immunological roles of IRAK1, including the relationship between IRAK1 and prognosis, genetic mutation, GO and KEGG enrichment pathway analysis, immune state of different tumors, The results showed that IRAK1 levels were upregulated in more than 20 types of cancers compared to the normal tissues. IRAK1 expression was associated with poorer prognosis in different cancer types. For the most frequent DNA alteration of IRAK1 is amplification. And the result of the enrichment analysis suggested that IRAK1 related to immune checkpoint pathway in cancer. IRAK1 inhibitor pacritinib inhibit proliferation and upregulate PD-L1 expression in different cancer cell lines. Moreover, the patients who receiving anti-PD-L1 therapy with low IRAK1 expression had a better prognosis, and the objective response rate to anti-PD-L1 therapy was higher in the low IRAK1 group than in the high IRAK1 group in IMvigor210 cohort. Our study reveals that IRAK1 can function as a prognostic marker in various malignant tumors. And pacritinib upregulated PD-L1 expression in several cancer cell lines, which indicating that IRAK1 can be used as a reliable marker to predict the efficacy of immunotherapy.

## Introduction

Cancer is a complex disease with tumor heterogeneity and regulated by tumor immune microenvironment (Hanahan and Weinberg 2011). As an alternative to traditional anticancer therapies, emerging immune checkpoint inhibitors (ICIs) have been shown to be effective in multiple cancer types (12), such as anti-CTLA-4, anti-PD-L1, and anti-PD-1 (Galluzzi et al., 2020; Vaddepally et al., 2020). Anti-PD-1 inhibitors such as pembrolizumab, toripalimab, and nivolumab have been approved as first-line therapies for patients with unresectable melanoma, non-small cell lung cancer and kidney cancer (Khoja et al., 2015; Keam 2019; Zhao et al., 2020). Existing clinical studies have shown that only part of patients can benefit. Although studies have shown that tumor mutation burden (TMB), tumor microsatellite instability (MSI), tumor copy-number alterations (CAN) and PD-L1 expression level can be used to predict the prognosis of ICIs (Dudnik et al., 2018; Davis and Patel 2019; Samstein et al., 2019; Lu et al., 2020). However, these biomarkers exhibited certain limitations, for example, the expression levels of PD-L1 cannot be uniform at different tumor. Therefore, it is urgent to identify new markers to find more people suitable for ICIs.

Interleukin-1 receptor-associated kinases comprise a class of serine-threonine kinases, including IRAK1, IRAK2, IRAK3, and IRAK4 (Flannery and Bowie 2010; Rhyasen and Starczynowski 2015). Previous studies suggested that the IRAK family is key to regulating inflammatory, innate immunity, and metabolic diseases (Su, Xu, and Huang 2020). IRAK1 is an active kinase that plays a critical role in the IL-1/TLR signaling pathway involved in inflammation and innate immune responses (Vollmer et al., 2017; Singer et al., 2018). In recent years, IRAK1 expression or alteration has been reported in several cancers. For instance, Wee et al. (Wee et al., 2015) reported that IRAK1 is overexpressed in breast cancers and that IRAK1 inhibition reduces cancer proliferation and metastasis. In addition, Liu et al. showed that IRAK1 contributed to chemoresistance in nasopharyngeal carcinoma through the IRAK1-S100A9 axis (Liu et al., 2021). Interestingly, IRAK1 overexpression was also observed in hepatocellular carcinoma, augmenting cancer stemness and drug resistance (Cheng et al., 2018). Given the potential role of IRAK1 in tumorigenesis, it is essential to conduct a pan-cancer analysis of it.

Moreover, existing papers have not fully elucidated the role of IRAK1 in adaptive immune responses. Thus, our study investigated the potential molecular and immune-related pathways of IRAK1 in various cancer types. We found that IRAK1 is related to immune pathways, including the PD-L1 and PD-1 checkpoint pathway in cancer. Furthermore, inhibitors of IRAK1 pacritinib upregulated PD-L1 expression in several cancer cell lines, indicating that the pharmacological inhibition of IRAK1 could be synergistic with immunotherapy in the future.

## Methods

### Data Processing and Expression Analysis

All original data were downloaded from The Cancer Genome Atlas (TCGA) (http://cancergenome.nih.gov/).We used GEPIA database to evaluate the expression of tumor tissues and the normal control of the TCGA data. The violin plots of the IRAK1 expression in different pathological stages were also obtained in the GEPIA database. Additionally, the correlation between the OS and PFS survival and the expression of IRAK1 was evaluated through the “Survival Analysis” module of GEPIA2. The cut-off valued was determined automatically (high expression (50%) vs. low expression (50%)). A log *p*-value < 0.05 was considered statistically significant.

The total protein expression of IRAK1 in tumor and normal tissues were explored in the UALCAN portal (http://ualcan.path.uab.edu/analysis-prot.html). The datasets of breast cancer, ovarian cancer, clear cell RCC, UCEC and LUAD are available.

The immunohistochemistry (IHC) images of IRAK1 protein expression in normal tissues and tumors tissues, including breast cancer, colorectal cancer, low-grade glioma and ovarian cancer, were downloaded from the HPA (http://www.proteinatlas.org/).

### Genetic Mutation Analysis

The frequencies of IRAK1 copy number alterations and mutations were identified in the cBioPortal (http://cbioportal.org), which is an open-access resource. The correlation of IRAK1 mutations and the survival in LSCC was also obtained.

### IRAK1- Related Genes Enrichment Analysis

IRAK1 binding proteins related genes were obtained on the STRING website (http://string-db.org/). IRAK1 correlated genes were obtained through the GEPIA2. Then the top five genes which exhibited the most significant correlation were determined and the TIMER2 was used to display the heatmap between the selected five genes and the IRAK1 expression across the pan-cancer types. The partial correlation and *p*-value was adjusted in the purity spearman’s rank correlation test.

The GO and KEGG enrichment pathway analysis were applied to the “clusterProfiler” R package by using the R software (3.6.3 version). Moreover, we used the “ggplot2” R packages for visualization.

### Immune-Infiltration Analysis and the IRAK1 Expression Data With Immunotherapy

The heatmaps of correlation between immune-suppressive genes and IRAK1 expression levels were shown by TIMER2.0. The CD274 and IRAK1 mRNA expression correlation was evaluated in different cancer types from the TCGA database using the TIMER2.0 tool. CIBERSORT and TIMER algorithms calculated the putative proportion of the different immune cells and correlated with IRAK1 expression in pan-cancer types. We analyzed IRAK1 expression in different immune subtypes in TISIDB (http://cis.hku.hk/TISIDB/index.php). To determine whether IRAK1 expression predicts the benefits of immunotherapy, we downloaded the IMvigor210 information from the http://research pub.gene.com/IMvigor210CoreBiologies, which include 298 patients with complete clinical data for urothelial carcinoma.

### Cell Culture

All cell lines, including MDA-MB-231, U251, Hep3B, Kyse30 and A498 were purchased from ATCC. MDA-MB-231, U251 and Hep3B cells were cultured in Dulbecco’s Modified Eagle Medium (DMEM) supplemented with 10% fetal bovine serum. Meanwhile, Kyse30 and A498 cells were cultured in RPMI 1640 supplemented with 10% fetal bovine serum.

## Results

### IRAK1 Expression in Pan-Cancer From the TCGA Database

**FIGURE 1:**
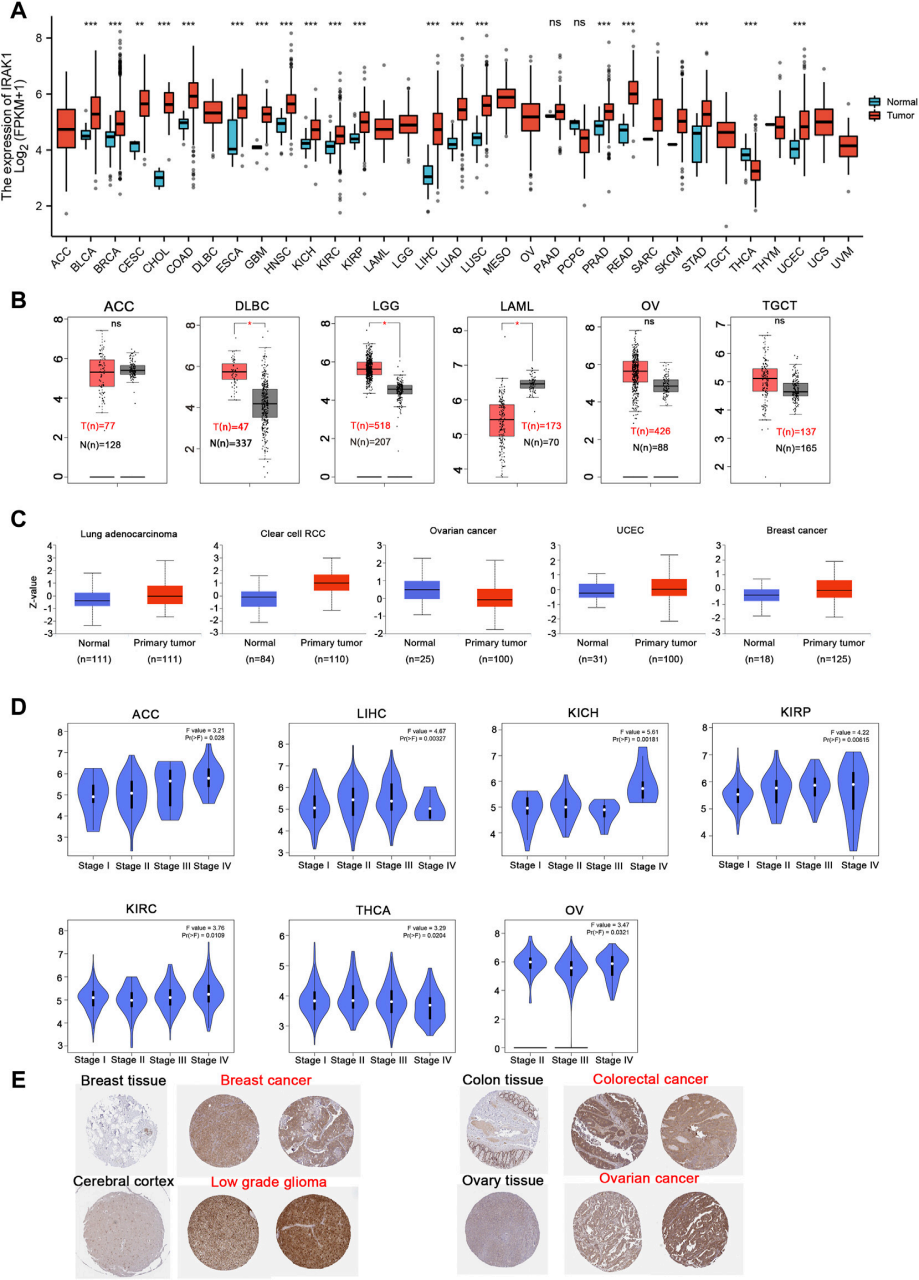
Expression level of IRAK1 in different cancer and pathological stages. **(A)** IRAK1 expression in different cancer types from the TCGA database analyzed by the TIMER database. *p < 0.05; **p < 0.01; ***p < 0.001. **(B)** IRAK1 expression in the cancer type of ACC, DLBC, LGG, LAML, OV, TGCT from TCGA database analyzed by GEPIA database, and the normal tissues were included as controls. **p < 0.01. **(C)** The expression level of IRAK1 total protein between normal tissue and primary tissue of lung adenocarcinoma, clear cell RCC, ovarian cancer, UCEC and breast cancer were analyzed from the CPTAC dataset. ***p < 0.001. **(D)** The expression of IRAK1 based on the different pathological stages were analyzed in ACC, LIHC, KICH, KIRP, KIRC, THCA, OV in the TCGA database. **(E)** Representative immunohistochemical staining of IRAK1 in breast cancer, colorectal cancer, low grade glioma and ovarian cancer from the HPA (http://www.proteinatlas.org/).

We analyzed IRAK1 mRNA expression in tumor and normal tissue samples across 33 pan cancers from the TCGA database. The results indicated that IRAK1 levels upregulated in BLCA, BRCA, CESC, CHOL, COAD, ESCA, GBM, HNSC, KICH, KIRC, KIRP, LIHC, LUAD, LUSC, PRAD, READ, STAD, UCEC, DLBC, and LGG compared to their corresponding normal tissues (*p* < 0.05) (Figures 1A,B). However, IRAK1 expressions downregulated in THCA and LAML compared to those in normal tissues. We further evaluated the total protein levels of IRAK1 from the CPTAC dataset; the results revealed higher expressions in the primary tumor of LUAD, clear cell RCC, UCEC, and breast cancer than in normal tissues (Figure 1C). In addition, through GEPIA2.0, we found that IRAK1 levels were significantly different in different pathological cancer stages, including ACC, LIHC, KIRP, KIRC, THCA, and OV (Figure 1D). Specifically, higher expressions correlated with higher stages in ACC, KICH, KIRP, and KIRC.

Then, we investigated IRAK1 protein expressions from the HPA database, which provided the IHC results of IRAK1 expression in tumor and normal tissues. The analysis showed that normal breast, colon, cerebral cortex, and ovary tissues exhibited a negative or weak staining of IRAK1, while in the corresponding tumor tissues, such expressions displayed moderate or strong staining (Figure 1E). Altogether, these results indicated that IRAK1 might play a significant role in different cancers.

### IRAK1 as a Prognostic Biomarker in Multiple Cancers

**FIGURE 2:**
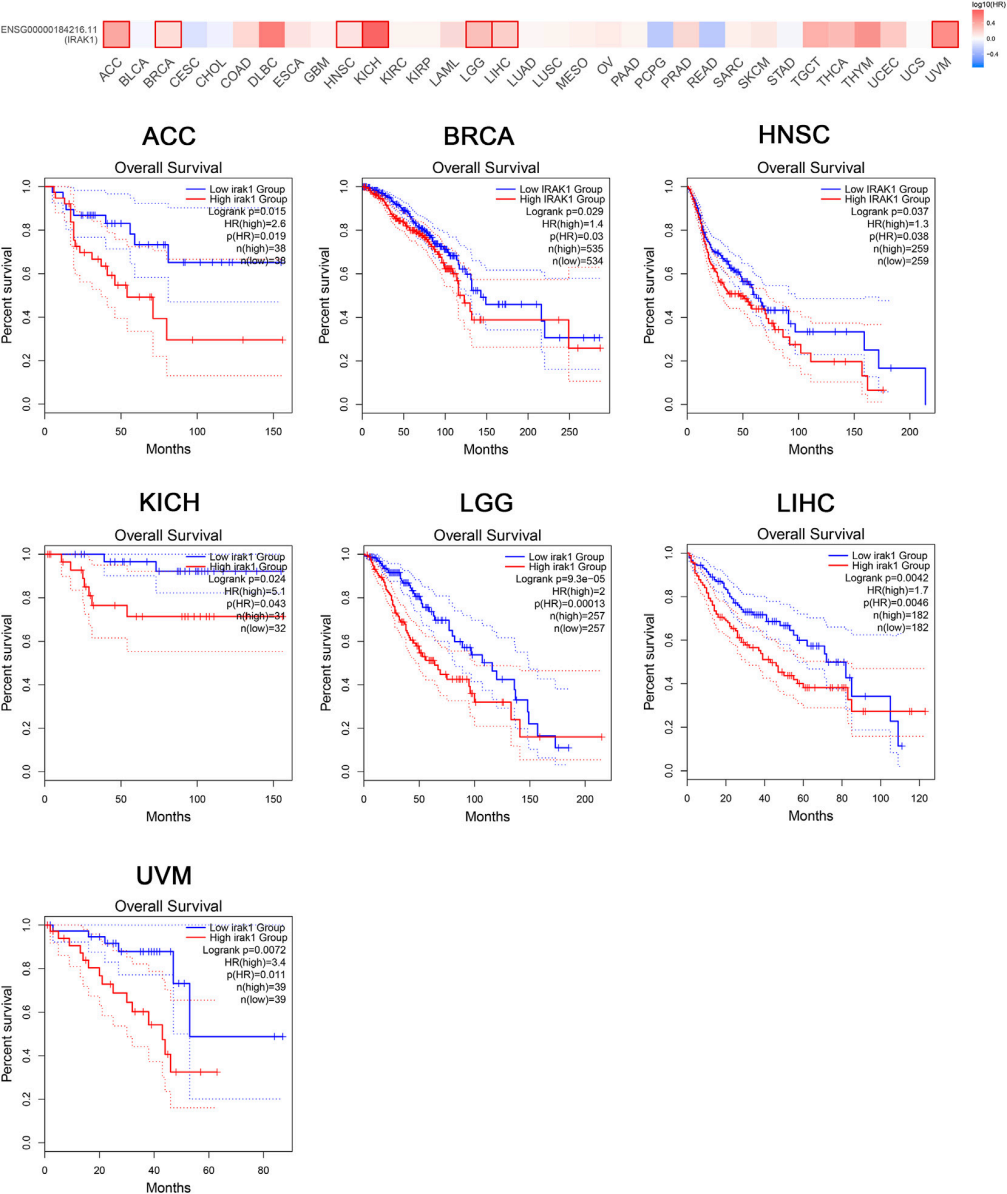
Kaplan-Meier overall survival curve of pan-cancers with high and low expression of IRAK1. Correlations between IRAK1 gene expression and overall survival from TCGA database are showed. The GEPIA2 tool was used to show the survival map and perform the survival curves.

The prognostic value of IRAK1 in most human cancers remained unknown. Thus, we aimed to evaluate the prognostic role by analyzing the TCGA cohort using GEPIA2.0. We found that IRAK1 expression was associated with a poorer overall survival (OS) in ACC (*p* = 0.015, HR (high) = 2.6), BRCA (*p* = 0.029, HR (high) = 1.4), HNSC (*p* = 0.037, HR (high) = 1.3), KICH (*p* = 0.024, HR (high) = 5.1), LGG (*p* < 0.01, HR (high) = 2), LIHC (*p* = 0.0042, HR (high) = 1.7), and UVM (*p* = 0.0072, HR (high) = 3.4) (Figure 2).

**FIGURE 3:**
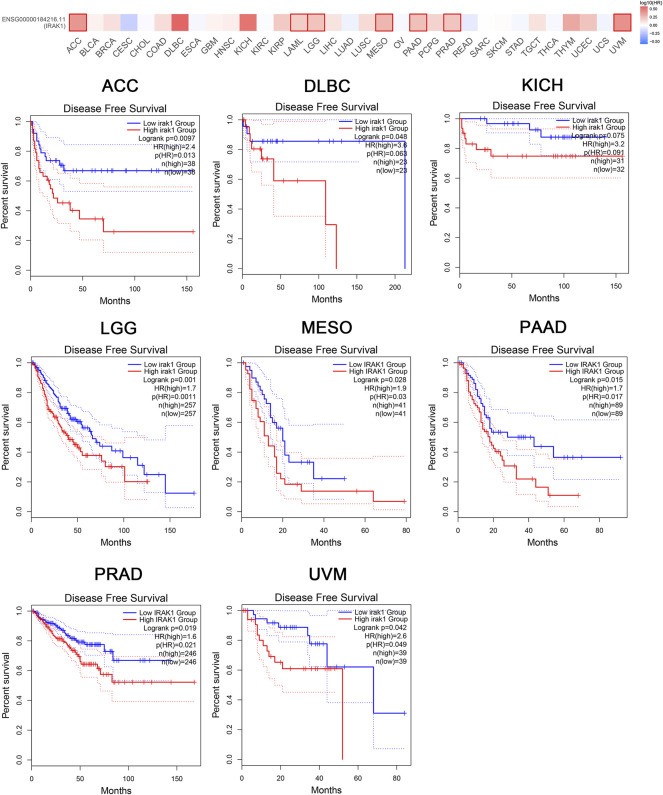
Kaplan-Meier disease-free survival curve of pan-cancers with high and low expression of IRAK1. Correlations between IRAK1 gene expression and disease-free survival from TCGA database are showed. The GEPIA2 tool was used to show the survival map and perform the survival curves.

Moreover, the analysis of DFS data revealed associations between high IRAK1 expression and poor prognosis among patients with ACC (*p* = 0.0097, HR (high) = 2.4), DLBC (*p* = 0.048, HR (high) = 3.6), KICH (*p* = 0.075, HR (high) = 3.2), LGG (*p* = 0.001, HR (high) = 1.7), MESO (*p* = 0.028, HR (high) = 1.9), PAAD (*p* = 0.015, HR (high) = 1.7), PRAD (*p* = 0.019, HR (high) = 1.6), and UVM (*p* = 0.042, HR (high) = 2.6) (Figure 3). We further conducted analysis of DSS and PFS data across different cancers by showing forest plots (Supplementary Figure S1). The above data indicated IRAK1 as a potential prognostic biomarker in multiple cancers.

### Frequencies of IRAK1 Alteration in Multiple Cancers

**FIGURE 4:**
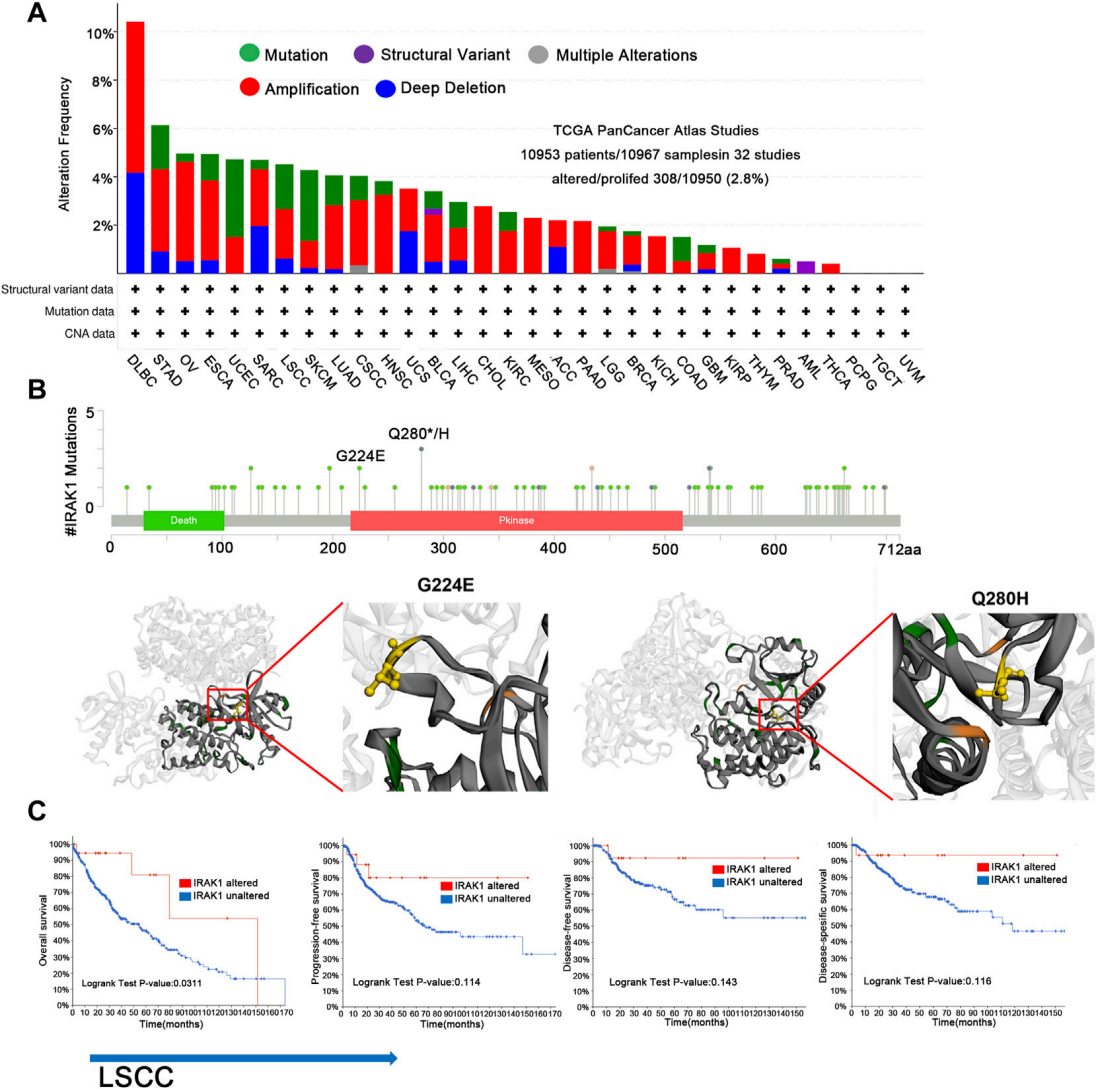
Genetic aberration of IRAK1 in human pan-cancers from TCGA database. **(A)** Display of genetic aberration of IRAK1 by using the cBioportal tool. The alteration frequency was 2.8% (10,953 patients/10967 samples in 32 studies from TCGA pan-cancer panel). **(B)** All mutation sites of IRAK1 were distributed using cBioPortal. The most frequent mutation was Q180H/* in Pkinase site. Another mutation named G224E was also displayed. **(C)** Correlations of mutation status and different survival status of LSCC are shown using cBioPortal.

We then curated a pan-cancer analysis of IRAK1 genetic alteration. In the TCGA pan-cancer panel, the most frequent DNA alteration was amplification. Mutations were likewise distributed in multiple cancers, including STAD, ESCA, UCEC, LSCC, and SKCM (Figure 4A). The most frequent mutation was Q180H/*, situated in the Pkinase site. Another frequent mutation was G224E (Figures 4B). We then determined the correlation between mutations and prognosis in LSCC. We found that cases with altered IRAK1 depicted a better prognosis in overall survival (*p* = 0.311) and exhibited a trend of prognostic value in progression-free survival (*p* = 0.114), disease-free survival (*p* = 0.143), and disease-specific survival (*p* = 0.116) compared to cases without altered IRAK1 (Figure 4C). These results indicated that a high IRAK1 expression in tumor tissue might be due to gene amplification. Additionally, IRAK1 mutation might reduce its role in cancer, requiring further exploration.

### Association of IRAK1 With Immune-Related Pathways Through Enrichment Analysis

**FIGURE 5:**
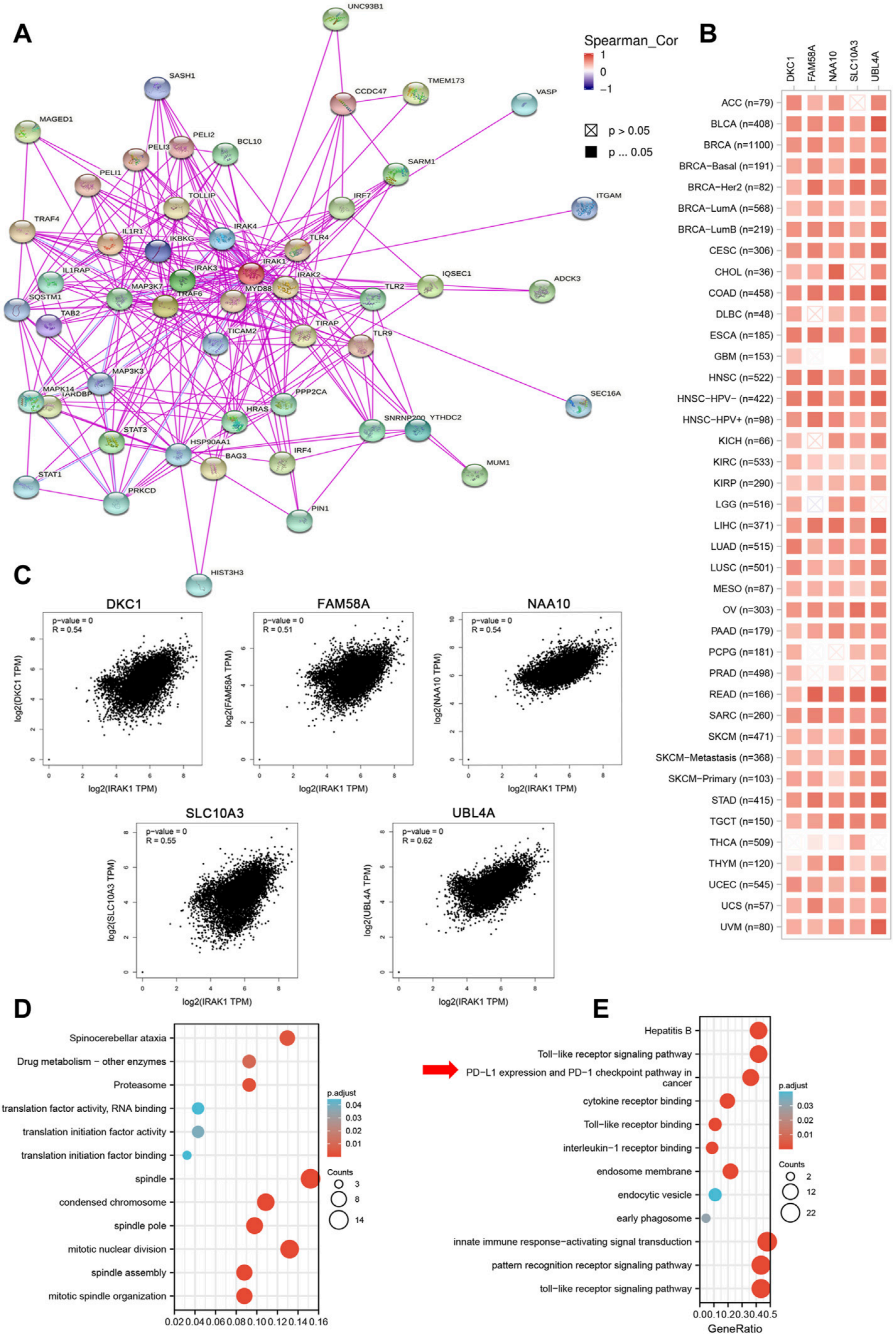
IRAK1-related gene enrichment analysis. **(A)** IRAK1 binding proteins were determined by using STRING tool. **(B)** Top 100 IRAK1 correlated genes were analyzed by using GEPIA2 tool and the correlation of IRAK1 and top five genes (DKC1, FAM58A, NAA10, SLC10A3, UBL4A) were shown. **(C)** The correlation map of IRAK1 and top five correlated genes in TCGA pan-cancer panel was analyzed by using TIMER2.0. **(D)** GO-KEGG pathways analysis was performed based on the IRAK1 correlated genes. **(E)** GO-KEGG pathways analysis was performed based on the IRAK1 binding genes.

We attempted to investigate the molecular mechanism of IRAK1 through analyzing the related and binding genes, then performed the enrichment analysis. As shown in Figure 5A, the interactions of IRAK-binding proteins were depicted using the STRING tool. We identified the top five related genes (DKC1, FAM58A, NAA10, SLC10A3, UBL4A) that mostly correlated with IRAK1 by GEPIA2.0; the heatmap displayed the correlation in pan-cancers (Figure 5B). In the TCGA pan-cancer cohort, IRAK1 expression positively correlated with DKC1 (R = 0.54), FAM58A (R = 0.51), NAA10 (R = 0.54), SLC10A3 (R = 0.55), and UBLA4 (R = 0.62) (Figure 5C). Notably, the KEGG and GO enrichment analysis of IRAK1 correlated genes suggested that IRAK1 was related to immune pathways, including PD-L1 and PD-1 checkpoint pathway in cancer, Toll-like receptor signaling pathway, interleukin-1 receptor binding, and innate immune response-activating signal transduction (Figures 5D,E).

### Correlations Between IRAK1 and Immune Checkpoint-Associated Genes and TME

**FIGURE 6:**
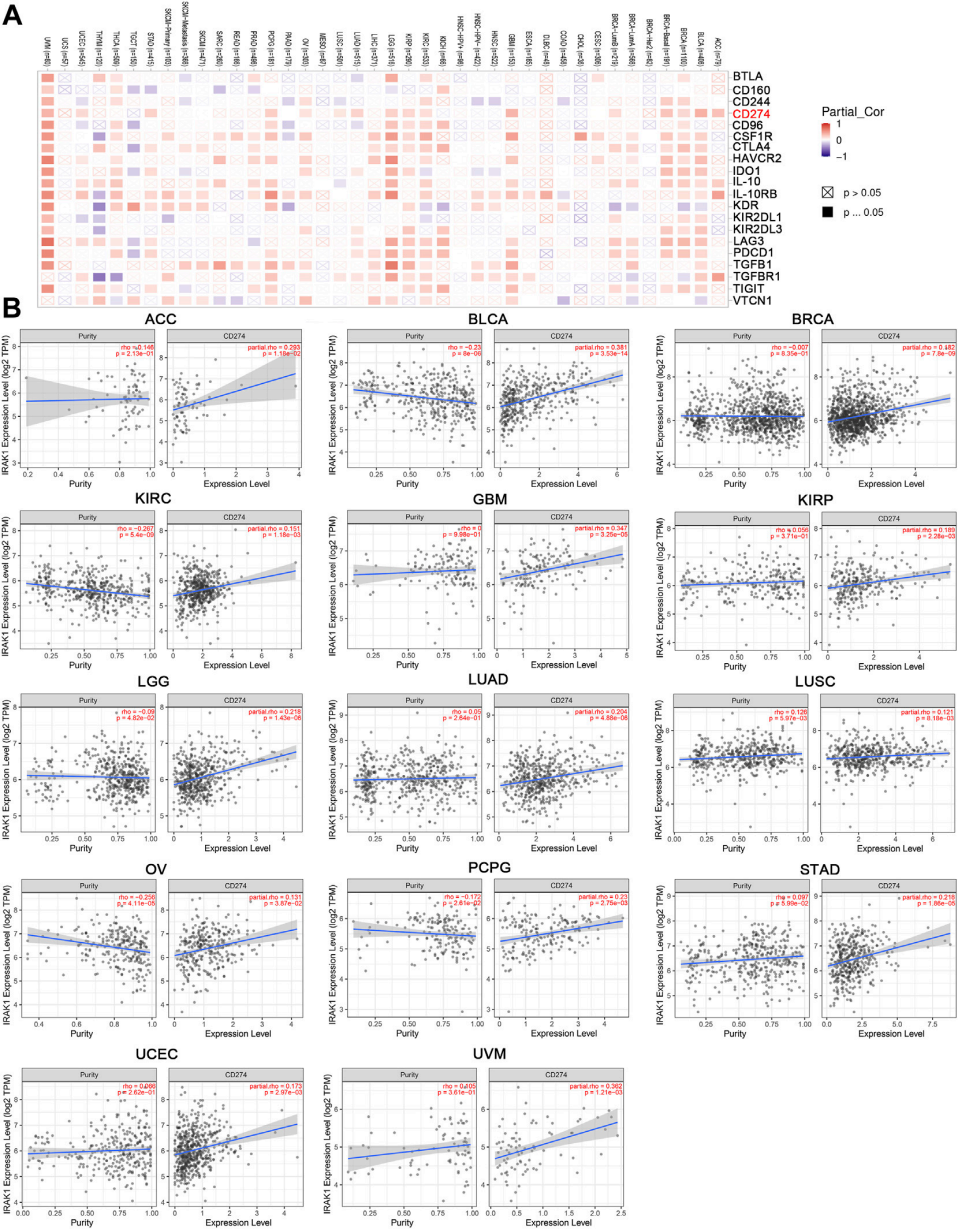
IRAK1 expression is associated with immune checkpoint-associated genes in pan-cancers. **(A)** Heatmap representation of the correlation between IRAK1 expression and immune checkpoint-associated genes across pan-cancer types. **(B)** IRAK1 expression was positive associated with CD274 expression in ACC, BLCA, BRCA, KIRC, GBM, KIRP, LGG, LUAD, LUSC, OV, PCPG, STAD, UCEC and UVM analyzed by using TIMER2.0.

Previous studies have demonstrated that immune checkpoint genes significantly influence tumor microenvironment and response to immunotherapy. Thus, we investigated the associations of IRAK1 expression and 20 primary immune checkpoint genes. The results exhibited strong positive relations with IRAK1 expression in multiple cancer types, including BLCA, BRCA, GBM, KIRC, KIRP, LGG, PCPG, and UVM, suggesting that a high IRAK1 expression might predict the better therapeutic efficacy of immunotherapy in targeting immune checkpoint genes (Figure 6A). KEGG and GO enrichment analysis of IRAK1 correlated genes suggested that IRAK1 was related to immune pathways, including PD-L1 and PD-1 checkpoint pathways. We further explored the potential relationship between PD-L1 (CD274) and IRAK1 gene expression in diverse cancer types of TCGA. A positive statistical correlation existed in ACC, BLCA, BRCA, KIRC, GBM, KIRP, LGG, LUAD, LUSC, OV, PCPG, STAD, UCEC, and UVM (Figure 6B).

**FIGURE 7:**
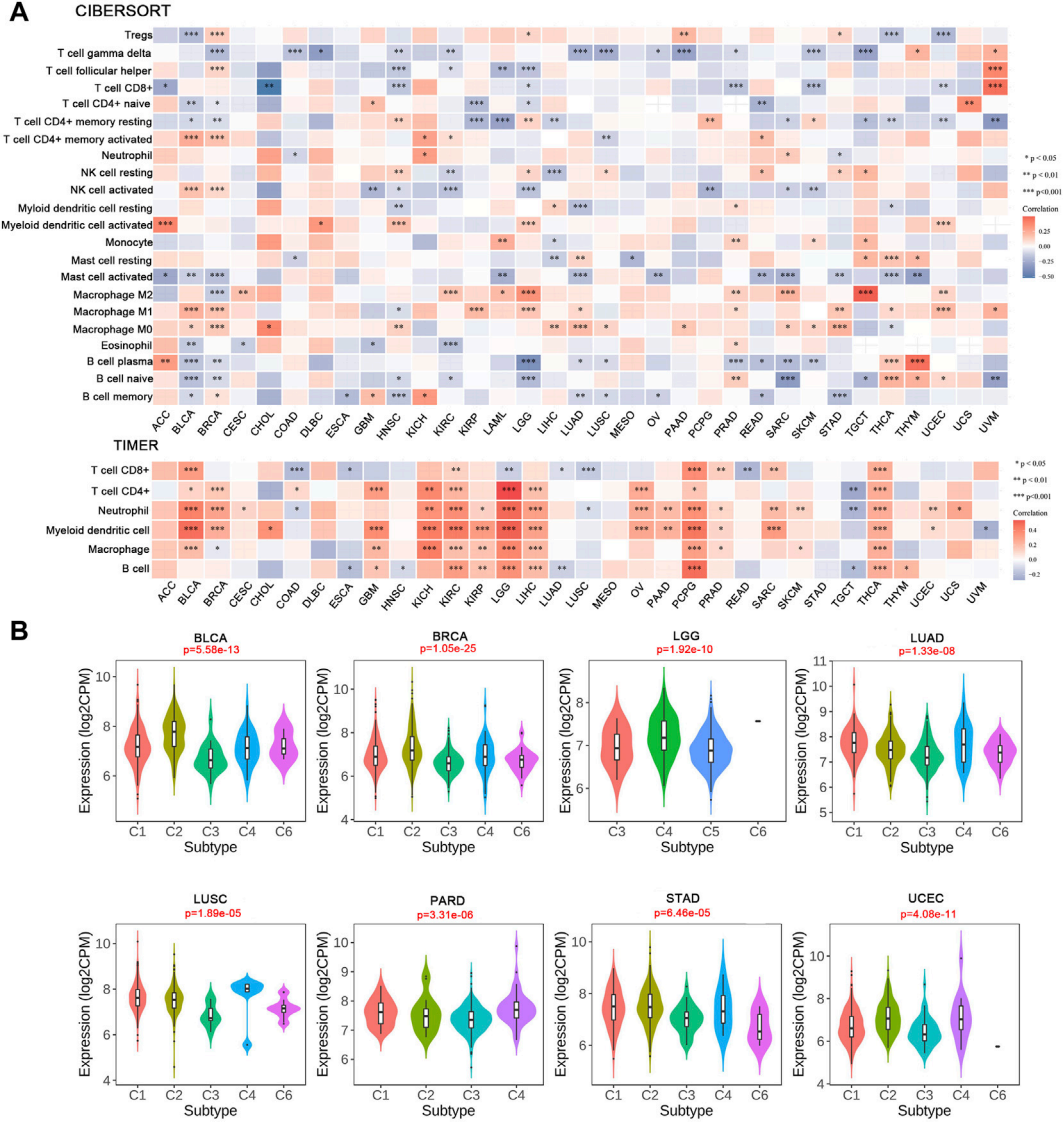
Correlation analysis between IRAK1 expression and tumor-infiltrating immune cells. **(A)** Correlation analysis of IRAK1 mRNA expression with different immune cells from TCGA database by CIBERSORT and TIMER. **(B)** The relationship between IRAK1 expression in pan-cancer immune subtypes. C1 (wound healing); C2(IFN-gamma dominant); C3 (inflammatory); C4 (lymphocyte depleted); C5 (immunologically quiet); C6 (TGF-b dominant).

After defining the associations with ICPs, we examined the relationship between IRAK1 and tumor-infiltrating immune cells in cancers using the CIBERSORT algorithm. The results revealed that IRAK1 expression negatively correlated with T cell CD8^+^ in seven cancer types, but a positive relationship in UVM. Conversely, a statistically positive relationship existed with myeloid dendritic cells activated in five cancer types, macrophage M2 in eight cancer types, and macrophage M1 in ten cancer types (Figure 7A). The TIMER algorithm analyzed the relationship between IRAK1 and immune-infiltrating cells for further validation. We further found a strong positive relationship with myeloid dendritic cells and macrophages in multiple cancers from TCGA (Figure 7A).

Afterward, we explored the IRAK1 mRNA levels in various immune subtypes using TISDB. Immune subtypes had six types, including C1 (wound healing), C2 (IFN-gamma dominant), C3 (inflammatory), C4 (lymphocyte depleted), C5 (immunologically quiet), and C6 (TGF-b dominant). IRAK1 expression differed significantly in immune subtypes in BLCA, BRCA, LGG, LUAD, LUSC, PARD, STAD, and UCEC (Figure 7B). However, no significant difference existed in other cancer types (data not shown). Altogether, these results suggested that IRAK1 significantly influenced the tumor microenvironment and might be a potential target for PD-1 antibody immunotherapy.

### IRAK1 Inhibitors Inhibit Proliferation and Upregulate PD-L1 Expression in Different Cancer Cell Lines

**FIGURE 8:**
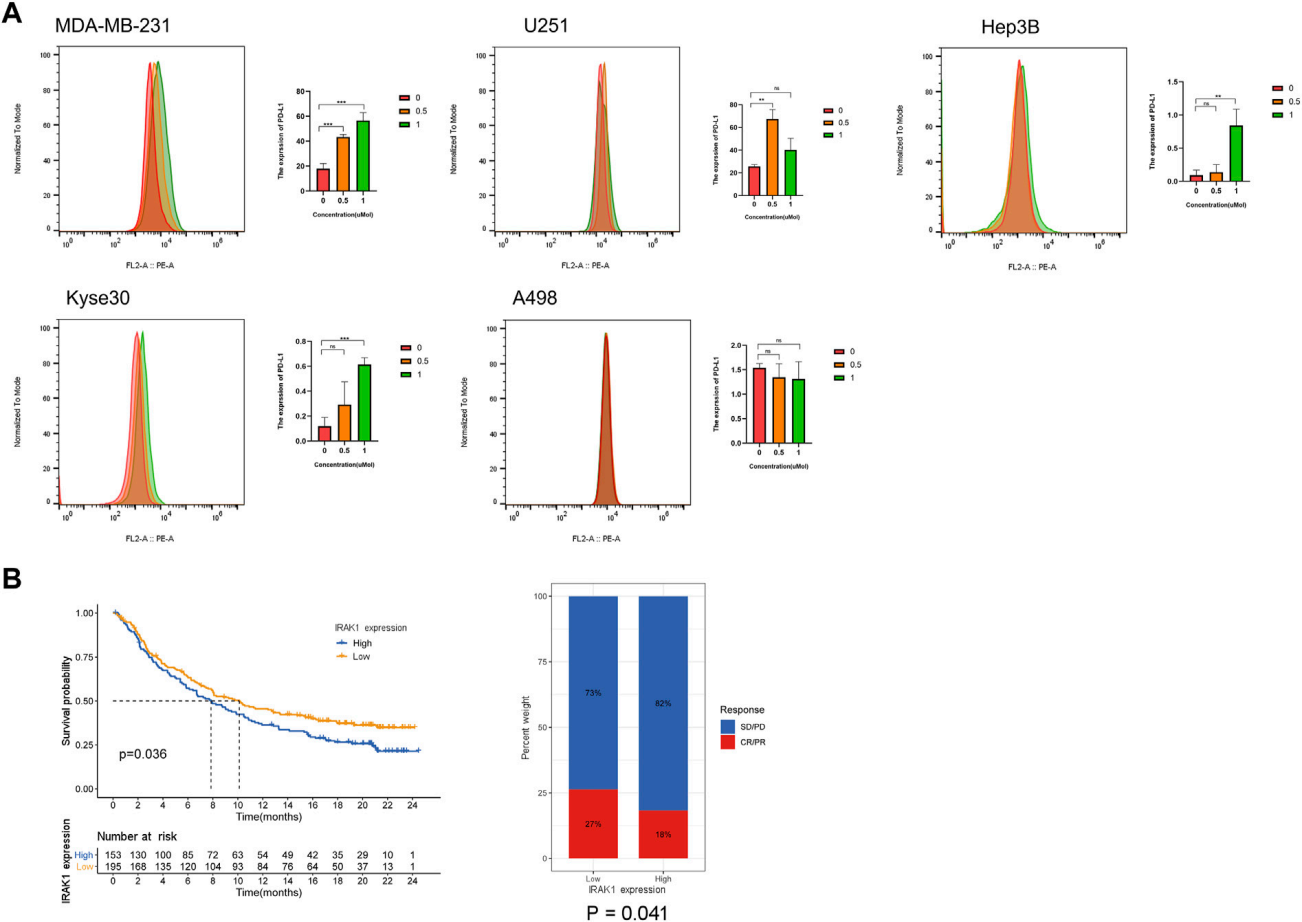
IRAK1 expression predicts the benefits in the immunotherapeutic treatments. **(A)** MDA-MB-231, U251, Hep3B, Kyse30 and A498 cells treated with DMSO control and IRAK1 inhibitors pacritinib (0.5µM, 1 µM) for 48 h were subjected to FACs analysis for cell surface PD-L1 expression. Quantification of PD-L1 is shown. Every experiment was run in three independent experiments. *<0.05, **<0.01, ***p < 0.001. **(B)** Patients receiving anti-PD-L1 immunotherapy in the IMvigor210 cohort were assigned high or low IRAK1. Notably, in the IMvigor210 cohort, patients with low IRAK1 had significantly better outcomes than those with high IRAK1 (*p* = 0.036). In the IMvigor210 cohort, the objective response rate to anti-PD-L1 therapy was higher in the low IRAK1 group than in the high IRAK1 group (27 vs. 18%, *p* = 0.041). The IMvigor210 dataset was downloaded from a freely available, fully documented software and data package, under the Creative Commons 3.0 license that can be downloaded from http://research-pub.gene.com/IMvigor210CoreBiologies. A total of 298 urothelial cancer cases with complete clinical information, were analyzed to determine the immunotherapy response.

Then, we evaluated the effect of IRAK1 inhibitor pacritinib on the growth and proliferation of different cancer lines by the MTT assay, including MDA-MB-231, U251, Hep3B, Kyse30, and A498. The results suggested that pacritinib inhibited the proliferation in different cancer lines. The mean IC50 values ranged from 0.789 to 2.612 μM (Supplementary Figure S2A). Additionally, the colony formation assay revealed the inhibitory effect of pacritinib in a dose-dependent manner (Supplementary Figure S2B). Finally, to further explore the relationship between the treatment of IRAK1 inhibitor and PD-L1 expression in cancer, we performed a flow cytometry assay to detect PD-L1 levels in different cancer types. The results indicated that pacritinib treatment significantly upregulated protein expressions of PD-L1 in MDA-MB-231, U251, Hep3B, and Kyse30 (*p* < 0.001). However, the expression of PD-L1 in A498 had not significantly changed post-treatment (Figure 8A).

### The Role of IRAK1 in the Prediction of Immunotherapeutic Benefits

In subsequent analyses, we examined the utility of IRAK1 expression the prediction of immunotherapeutic benefits. For the purposes of the study, patients receiving anti-PD-L1 immunotherapy in the IMvigor210 cohort were assigned high or low IRAK1. Notably, in the IMvigor210 cohort, patients with low IRAK1 had significantly better outcomes than those with high IRAK1 (*p* = 0.036). In the IMvigor210 cohort, the objective response rate to anti-PD-L1 therapy was higher in the low IRAK1 group than in the high IRAK1 group (27 vs. 18%, *p* = 0.041) (Figure 8B). Overall, these results indicate that the level of IRAK1 in cancer which could be significant biomarker both for predicting cancer survival and immunotherapy response in ACC, KICH, BRCA, LGG, and UVM.

## Discussion

IRAK1 plays a vital role in oncogenesis and tumor progression in multiple cancers and is shown to contribute to the progression of various cancers, including hepatocellular carcinoma (Chen et al., 2020), breast cancer (Wee et al., 2015), endometrial cancer (Wang et al., 2018), non-small cell lung cancer (Behrens et al., 2010) and melanoma (Boukerche et al., 2004; Srivastava et al., 2012). However, the systematic analysis in pan cancer and the relationship with immune system has not been investigated in detail.

In our study, we initially used GEPIA2, TIMER, and HPA databases to determine the mRNA, protein expression level of IRAK1 in cancers compared normal tissues, and found that its expression was significantly higher in 20 tumours. In addition, the expression level of IRAK1 was positively correlated with tumor stage in ACC, KICH, KIRP, and KIRC, suggesting that IRAK1 plays an important role in predicting tumor malignancy and aggressiveness. Then, the relationship between IRAK1 expression and prognosis were explored. The OS analysis indicated that IRAK1 is a risk factor for patients with ACC, BRCA, HNSC, KICH, LGG, LIHC and UVM. And for DFS, the results revealed that IRAK1 acts as a risk factor for patients with ACC, DLBC, KICH, LGG, MESO, PAAD, PRAD and UVM. These results indicated that IRAK1 is a potential prognostic biomarker and promotes oncogenesis and tumor progression in various cancer types, especially in the ACC and KICH.

We then presented the genetic alteration of IRAK1 across all cancer types in the TCGA cohort, depicting that the most frequent alteration was amplification. Although IRAK1 expression has been reported in several cancers to date, its amplification had not been examined before.

However, the relationship between IRAK1 and immune cells or immune pathways in tumors remains unclear. Therefore, we found that IRAK1 is related to a variety of immune signaling pathways in tumors, including toll-like receptor signaling Pathway, interlekin-1 receptor binding and innate immune response-activating signal transduction and immune checkpoint signaling. The relationship between IRAK1 and autoimmunity has been explored. For example, some studies have examined the potential associations of SNPs in IRAK1 and miRNA-146 and the development of arthritis (Chatzikyriakidou et al., 2010a; Chatzikyriakidou et al., 2010b; Song et al., 2015). Moreover, evidence suggested that anti-IRAK1 exhibited unusual activity in a murine arthritis model (Madan et al., 2012). Another study identified the genetic association between IRAK1 SNPs and the increased risk factors for SLE (Jacob et al., 2009). On the other hand, it has been reported that IRAK1 regulates immune cells to control excessive inflammatory responses *in vivo* and induce chronic inflammation by participating in TLR and Interlekin-1 receptor signaling pathways (Wang et al., 2020; Hu et al., 2021). Further analysis of the relationship between IRAK1 and tumor immune-infiltrating cells showed that IRAK1 was positively correlated with M2 macrophage cells and negatively correlated with CD8+T cells in multiple tumors. It is well-known that M2 tumor-associated macrophages inhibit immune cells against tumor immune responses, leading to the formation of tumor immunosuppressive microenvironment and promoting tumor proliferation and metastasis (Yang et al., 2020). In addition, IRAK1 have reported that promotes the progression of hepatocellular carcinoma by participating in the chronic inflammation mediated by macrophages, which is consistent with the positive correlation between IRAK1 and macrophage expression in liver cancer found in this study (Li et al., 2015).

To our knowledge, as anti- PD-1/PD-L1 had attained considerable clinical efficacy in various cancer types, the relationship between IRAK1 and PD-1/PD-L1 axis remains unknown, which motivated us to explore the role of IRAK1 in PD-L1 regulation. To understand fully the role of IRAK1 in the regulation of PD-L1 protein expression, we investigated the associations of IRAK1 expression and PD-L1 and other main immune checkpoint genes. The results indicated that strong positive relationships with PD-L1(CD274) and IRAK1 gene expression in diverse cancer types of TCGA. Using IMvigor210 to evaluate patients receiving anti-PD-L1 therapy, we found that patients with low IRAK1 expression had a better prognosis, and the objective response rate of patients with low IRAK1 expression was higher than that of patients with high IRAK1 expression, indicating that IRAK1 can be used as a reliable marker to predict the efficacy of immunotherapy. Overall, this suggests that immunotherapy may benefit patients with low IRAK1 expression.

Although IRAK1 has been proved to play an important role in tumor malignant proliferation, metastasis and drug resistance acquisition in a variety of tumors, the clinical usefulness of IRAK1 inhibitor has not been clarified in clinical studies (Jain, Kaczanowska, and Davila 2014; Wee et al., 2015; Meng et al., 2020). Pacritinib, an IRAK1 inhibitor, which has been shown to be effective in myelofibrosis, and acute myeloid leukemia (Hart et al., 2011; Mesa et al., 2017). We used pacritinib to treat five different tumor cell lines, including esophageal cancer, liver cancer, glioma, breast cancer and kidney cancer, and found that pacritinib effectively inhibited tumor proliferation, suggesting that pacritinib may be a potential anti-pan cancer inhibitor. In addition, pacritinib has also been reported to reduce chemotherapy resistance in nasopharyngeal carcinoma, mainly by regulating the phosphorylation level of IRAK1, thereby inhibiting the expression of S100A9 and reducing the patients with nasopharyngeal carcinoma resistance to paclitaxel (Liu et al., 2021). In order to verify the relationship between PD-L1 and IRAK1, we used flow cytometry to detect PD-L1 expression in tumor cells treated with different concentrations of pacritinib and found that the level of PD-L1 upregulated, suggesting that IRAK1 correlated with PD-1/PD-L1 axis and mediated immunosuppression. Thus, inhibiting the expression level of IRAK1 in the tumor microenvironment may improve anti-tumor immune responses. In addition, the combination of IRAK1 inhibitor with immunotherapy is expected to be a feasible treatment for patients with cancer with high IRAK1 expression. Pacritinib, a IRAK1 inhibitor which also dual affects the expression of JAK2 and FLT3. And JAK2 and FLT3 have been proved to be important molecules involved in the regulation of PD-L1 in different kind tumors (Prestipino et al., 2018; Brodská et al., 2019). Therefore, there may be differences in the detail mechanism of its regulating PD-L1 in different tumors, which needs to be validated by further research.

Although we performed a comprehensive and systematic analysis on IRAK1 and utilized different databases for verifying the role of IRAK1, there are some limitations in this study. First, the sequencing data from the different databases exhibited differences and lacked granularity and specificity, which might entail systematic bias. Second, *in vivo* and mechanistic experiments are necessary to prove our results on the potential functions of IRAK1, which can increase our research credibility. Finally, the mechanisms by which IRAK1 participates in immune regulation remain unknown, and the exact pathways require further study.

To conclude, more specific and clinical samples are necessary to identify the benefits of anti-IRAK1 in cancer survival. Therefore, prospective studies on targeting IRAK1 to anti-tumor immunotherapy are vital.

## Data Availability

The raw data supporting the conclusion of this article will be made available by the authors, without undue reservation.

## Glossary

ACC: adrenocortical carcinoma
BLCA: bladder urothelial carcinoma
BRCA: breast invasive carcinoma
CAN: tumor copy-number alterations
CESC: cervical squamous cell carcinoma and endocervical adenocarcinoma
CHOL: cholangiocarcinoma
COAD: colon adenocarcinoma
DMEM: dulbecco’s modified eagle medium
DLBC: lymphoid neoplasm diffuse large B-cell lymphoma
ESCA: esophageal carcinoma
GBM: glioblastoma multiforme
HNSC: head and neck squamous cell carcinoma
ICIs: immune checkpoint inhibitors
KICH: kidney chromophobe
KIRC: kidney renal clear cell carcinoma
KIRP: kidney renal papillary cell carcinoma
LGG: brain lower grade glioma
LIHC: liver hepatocellular carcinoma
LUAD: lung adenocarcinoma
LUSC: lung squamous cell carcinoma
MESO: mesothelioma
MSI: tumor microsatellite instability
OV: ovarian serous cystadenocarcinoma
PRAD: prostate adenocarcinoma
PAAD: Pancreatic adenocarcinoma
PCPG: pheochromocytoma and paraganglioma
RCC: renal carcinoma
READ: rectum adenocarcinoma
STAD: stomach adenocarcinoma
TCGA: the cancer genome atlas
TMB: tumor mutation burden
THCA: thyroid carcinoma
UVM: uveal melanoma
UCEC: uterine corpus endometrial carcinoma
