# Supplementary material for: A Pan-Cancer Analysis of IRAK1 Expression and Their Association With Immunotherapy Response
Source: Front Mol Biosci. 2022 May 20;9:904959. doi: 10.3389/fmolb.2022.904959 (PMC9163706; doi:10.3389/fmolb.2022.904959)
Supplement: Supplementary file 1 [file Table1.doc]

|  | PD-L1(%) |  |  |  |  |  |  |  |  |  |  |
| --- | --- | --- | --- | --- | --- | --- | --- | --- | --- | --- | --- |
| MDA-MB-231-0 | 13.49 | 20.89 | 19.87 |  | 13.11 | 16.78 | 15.4 |  | 12.3 | 14.56 | 14.25 |
| MDA-MB-231-0.5 | 45.4 | 43.46 | 41.41 |  | 46.6 | 40.46 | 43.41 |  | 40.6 | 42.49 | 41.24 |
| MDA-MB-231-1 | 58.79 | 49.22 | 61.59 |  | 58.45 | 56.67 | 60.37 |  | 62.4 | 59.77 | 58.78 |
|  |  |  |  |  |  |  |  |  |  |  |  |
|  |  |  |  |  |  |  |  |  |  |  |  |
| U251-0 | 26.37 | 26.85 | 23.59 |  | 20.17 | 20.65 | 24.46 |  | 20.17 | 20.65 | 24.46 |
| U251-0.5 | 58.38 | 69.38 | 74.54 |  | 55.42 | 60.89 | 64.67 |  | 55.42 | 60.89 | 64.67 |
| U251-1 | 28.61 | 45.34 | 46.79 |  | 27.12 | 44.87 | 48.56 |  | 27.12 | 44.87 | 48.56 |
|  |  |  |  |  |  |  |  |  |  |  |  |
|  |  |  |  |  |  |  |  |  |  |  |  |
| hep3B-0 | 0.16 | 0.009 | 0.11 |  | 0.1 | 0.079 | 0.08 |  | 0.06 | 0.059 | 0.07 |
| hep3B-0.5 | 0.2 | 0.21 | 0.008 |  | 0.3 | 0.26 | 0.018 |  | 0.36 | 0.16 | 0.18 |
| hep3B-1 | 0.89 | 1.06 | 0.58 |  | 1.09 | 0.96 | 0.79 |  | 1.12 | 0.86 | 1.02 |
|  |  |  |  |  |  |  |  |  |  |  |  |
| kyse30-0 | 0.12 | 0.19 | 0.05 |  | 0.13 | 0.11 | 0.06 |  | 0.1 | 0.09 | 0.15 |
| kyse30-0.5 | 0.22 | 0.5 | 0.16 |  | 0.41 | 0.2 | 0.26 |  | 0.38 | 0.24 | 0.17 |
| kyse30-1 | 0.67 | 0.61 | 0.56 |  | 0.68 | 0.69 | 0.6 |  | 0.58 | 0.67 | 0.54 |
|  |  |  |  |  |  |  |  |  |  |  |  |
|  |  |  |  |  |  |  |  |  |  |  |  |
| A498-0 | 1.62 | 1.45 | 1.56 |  | 1.67 | 1.58 | 1.68 |  | 1.22 | 1.35 | 1.46 |
| A498-0.5 | 1.03 | 1.57 | 1.43 |  | 1.23 | 1.16 | 1.59 |  | 1.87 | 1.02 | 1.54 |
| A498-1 | 1.13 | 1.09 | 1.72 |  | 1.02 | 1.23 | 1.83 |  | 1.45 | 1.48 | 1.49 |

Table S1: The raw data of all repeats for ecah cell line of flow analysis
