# Supplementary material for: A Pan-Cancer Analysis of IRAK1 Expression and Their Association With Immunotherapy Response
Source: Front Mol Biosci. 2022 May 20;9:904959. doi: 10.3389/fmolb.2022.904959 (PMC9163706; doi:10.3389/fmolb.2022.904959)
Supplement: Supplementary file 2 [file DataSheet1.docx]

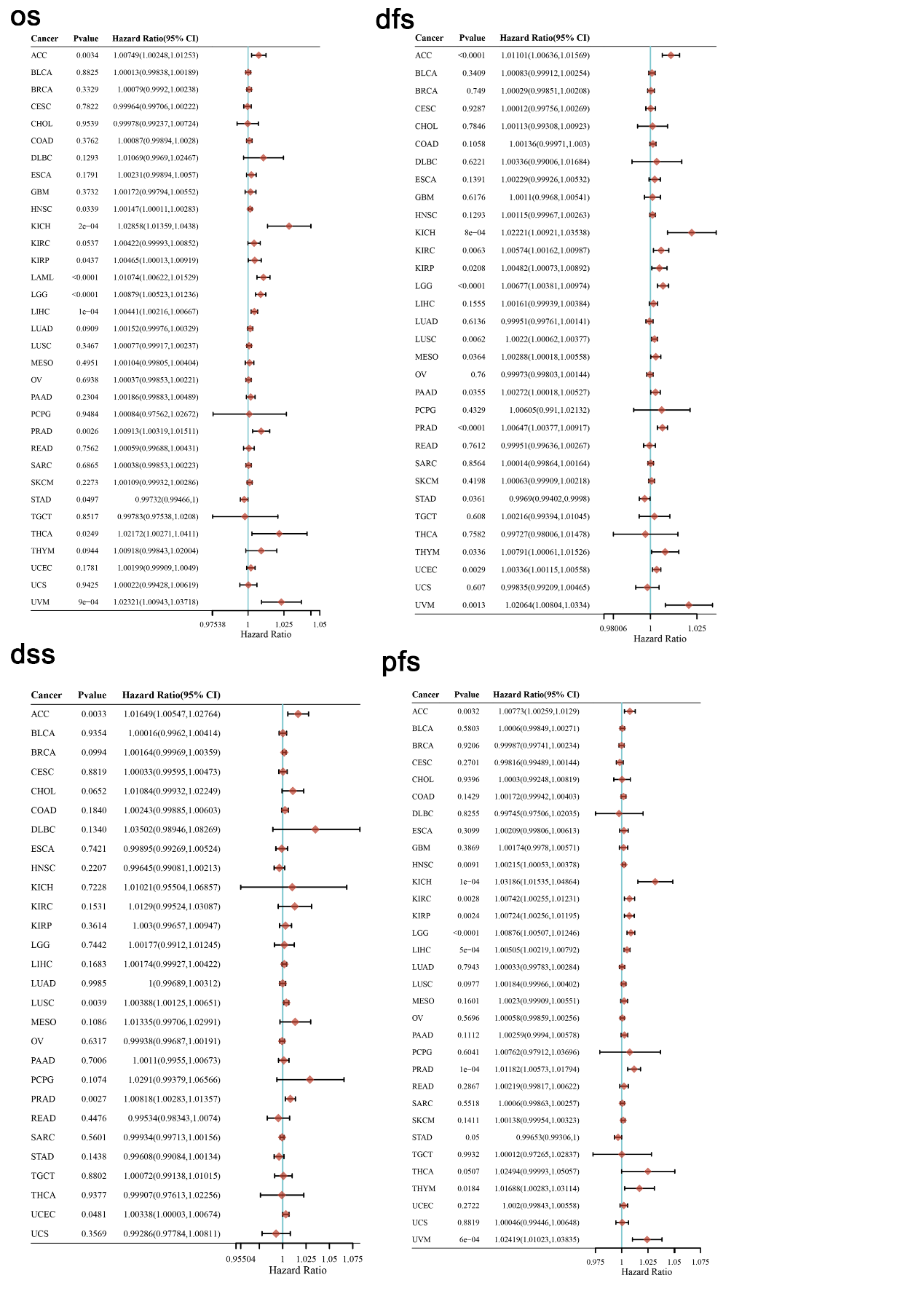


**Figure S1. Forest plots depict os, dfs, dss and pfs among cancer patients with low and high IRAK1 expression**


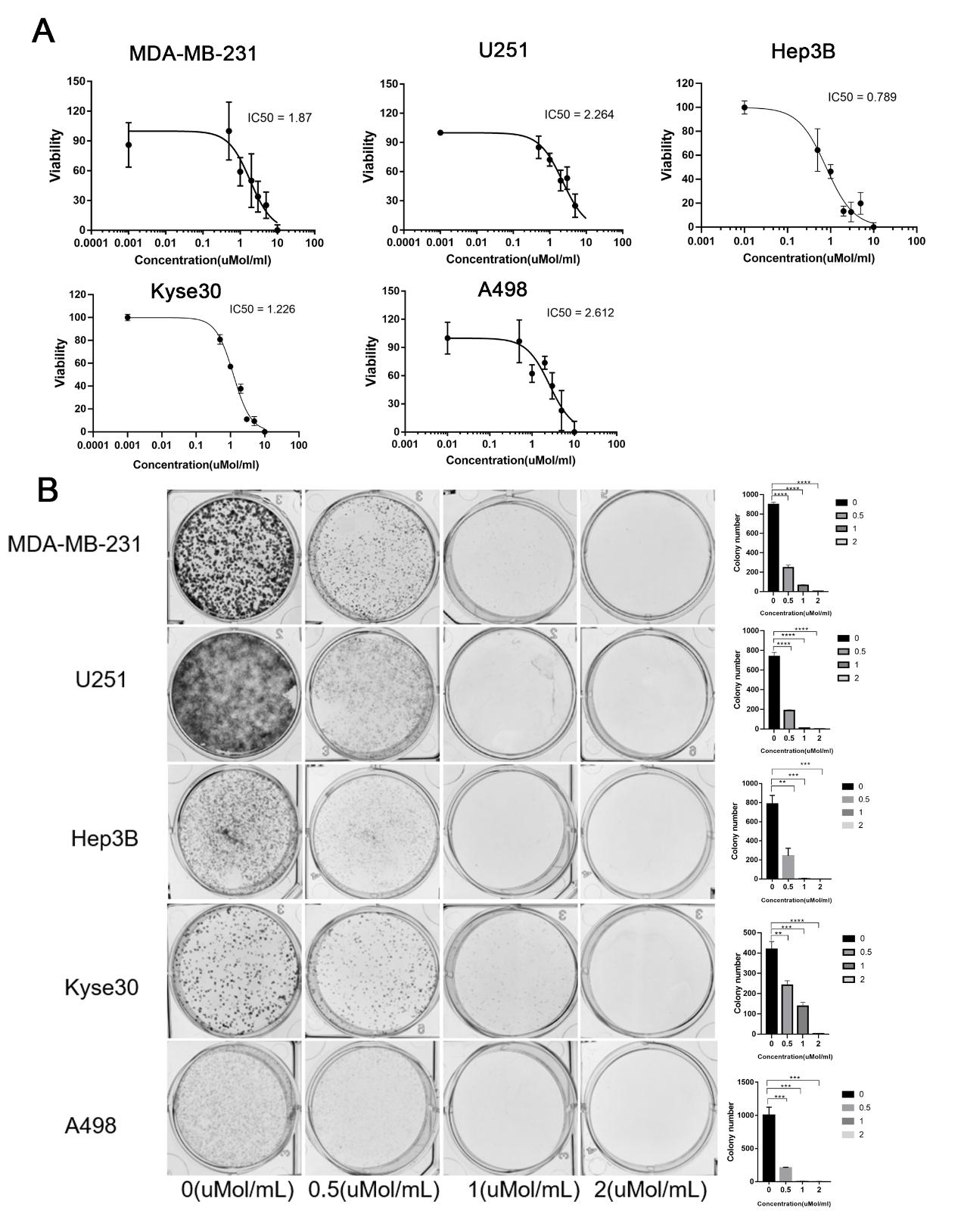


**Figure S2. Pacritinib inhibits proliferation of different cancer cell lines.** (A)Treat MDA-MB-231, U251, Hep3B, Kyse30 and A498 with different concentrations of pacritinib, and calculate the half-maximal inhibitory concentration (IC50) after 48 h (left). Data are shown as mean ± SD. The IC50 of 5 cancer cell lines are in the form of a bar graph (right). (B) Colony formation of MDA-MB-231, U251, Hep3B, Kyse30 and A498 under different pacritinib concentrations. The histogram indicates the number of clones (*P < 0.05, **P < 0.01, ***P < 0.001, ****P < 0.0001). Colony-forming units were visualized using a microscope and colonies containing >50 cells were enumerated.
